# Thermoformed Retainer: An Effective Option for Long-Term Stability

**DOI:** 10.1155/2020/8861653

**Published:** 2020-10-24

**Authors:** A. Giancotti, P. Mozzicato, G. Mampieri

**Affiliations:** Department of Orthodontics, University of Rome “Tor Vergata”, Rome, Italy

## Abstract

**Introduction:**

The concept of orthodontic retention is moving toward the idea that teeth will move unless retained indefinitely. However, permanent retention implies permanent supervision, and that is where reality clashes with stability. The cornerstone of Essix permanent retention is the complete delegation of responsibility to the patient. Essix retainers have nothing to adjust, but maximum collaboration of the patient is essential to achieve long-term stability. The purpose of this paper is to show the effectiveness of the thermoplastic appliance as a retainer, using a clinical case with a 10-year follow-up. *Case Report*. A 33-year-old male patient presented with a class II malocclusion division 2 and normal skeletal pattern. According to the patient's desire, a treatment plan was proposed to obtain the aesthetic result of the smile, maintaining the molar and canine class II relationship. The orthodontic therapy was performed by using the Invisalign System. In this case, it was possible to appreciate a posterior occlusal stability after 10 years.

**Conclusion:**

Currently, among orthodontists, the use of removable plastic devices is gaining popularity thanks to their capability to encapsulate and retain both posterior and anterior teeth. In this article, the technical features of thermoformed retainers will be described and one clinical case with a 10-year follow-up will be presented to emphasize the effectiveness of these retainers.

## 1. Introduction

In orthodontic treatment, there are some goals, and the stability of the correction achieved is one of the most important ones [1–4]. Retention of orthodontic treatment results and posttreatment stability are challenges for orthodontists. Although several factors affect stability, there are 3 major ones: time for reorganization of gingival and periodontal tissues, unstable position of the teeth after orthodontic treatment, and changes produced by growth [2, 5–8]. Our concerns about the stability of orthodontic treatment still seem to be the same as those expressed by Calvin Case in 1920: “If there is one part of orthodontia more than another that is absolutely indispensable to the success of this specialty and its establishment upon a firm foundation as one of the arts and sciences, it is the permanent retention of regulated teeth…. what does this temporary pleasure and satisfaction to ourselves and our patients amount to, if we find in a few years that the very cases which create in us the greatest pride, are going back to their former malpositions and disharmonies, in spite of everything we have been able to do with retaining appliances.” [9]. Vanarsdall and White summed up the problem as follows: “Early in the development of orthodontics, a serious misconception evolved. Dentists and the public were led to believe that orthodontic treatment could result in teeth that were straight for a lifetime.” [10]. For this reason, the effect of various diagnostic and treatment factors on occlusal stability in both the short term and the long term has been extensively investigated [2, 4–6, 9, 11–24]. Littlewood et al. failed to establish any reliable guidelines regarding the efficacy of various retention protocols [16]. Gianelly and others have argued that the stability of orthodontic treatment can be improved by preserving a mandibular intercanine width [19–21, 25]. This means that any increase in mandibular intercanine dimension is inherently unstable [31–33]. Along the same lines, Blake and Bibby listed six major criteria for the stability of finished orthodontic cases:

1. The patient's pretreatment lower arch form should be maintained unaltered as much as possible
2. The original lower intercanine width should be maintained as much as possible, because expansion of the lower intercanine width leads to the most predictable of orthodontic relapse
3. Mandibular arch length decreases with time
4. The most stable position of the lower incisor is its pretreatment position; advancing the lower incisors can seriously compromise stability
5. Fiberotomy is an effective means of reducing rotational relapse
6. Lower incisor reproximation can improve long-term posttreatment stability [4]

Little et al. have shown that only 10% of patients have a clinically stable alignment in the long term. In the study conducted at the University of Washington, the authors concluded that orthodontic results are more likely to be unstable than stable, and the only way to ensure ongoing satisfactory alignment after treatment would be to provide retention for life [13]. Also, Vaden et al. have shown that after 15 years following the end of orthodontic treatment, there was a “minimal irregularity” in the occlusal alignment [14]. Only in the study conducted by Dyer et al., researchers have shown that the correction of maxillary crowding was relatively stable in the long term [15]. However, a number of authors agree that long-term stability is closely linked to the type of removable retention appliance [1, 2, 17, 26–28].

The removable retainers show advantages and disadvantages. We know two types of removable devices for posttreatment retention: Hawley-type retainers and thermoplastic retainers. Sauget et al. noted that traditional Hawley retainers allow greater settling and thus an improvement in posterior occlusal contacts, compared to full-coverage thermoplastic retainers [29]. However, this type of retainer requires the patient's compliance, and, in addition, different from expectations, removable retainers require adequate daily cleaning in order to guarantee proper hygiene levels [30]. The major advantage of thermoplastic retainers is aesthetics. Such retainers are clear and almost invisible, so many patients prefer them to conventional Hawley devices. However, like other removable retainers, thermoplastic devices rely on patient compliance. Their material is not as durable as a Hawley type, and more replacements are needed. Finally, thermoplastic retainers do not allow occlusal settling if they are extended back to the molars, often causing anterior open bite [17, 31]. According to literature, the retainer shall be designed as comfortable and easy to wear in order to maintain occlusal stability [1, 2, 17, 26]. For this reason, Sheridan et al. in 1993 described the first invisible device [26]. The Essix appliance is an aesthetic removable plastic device, retained by the anatomy of the teeth. It is practically invisible, rapidly manufactured, and not so expensive [1, 2, 5, 6, 11–17, 26–28]. Thermoplastic retainers are generally made of two classes of material: copolyester type “A” Endure and polypropylene or ethylene copolymer type “C+” Duraforce [17]. The Essix type “A” is obtained from plastic copolyester material, in which a 1 mm thick sheet of plastic (Scheudent type A) is thermically formed on the cast reducing its thickness to 0.65 mm [26, 27]. This type “A” material is generally more aesthetic because of better clarity, although it tends to tear and crack [17, 27]. For this reason, the manufacturers are creating a new generation of thermoplastic materials, including ACE and Duraclear to improve durability, clarity, and retention [17]. Such thermoforming process guarantees the posterior stability of the appliance, providing flexibility for ease of insertion and removal, and does not interfere with speech. Therefore, it is comfortable, aesthetic, and well accepted by patients, who can easily wear the appliance full-time. However, the thermoformed appliances are limited in terms of wear resistance and fitting. A precision fit is essential in any thermoformed application, not only on initial placement but also until the appliance is worn [28]. The fit depends on the adaptation of the retentive gingival undercuts to the contact points: if they are not well defined, the appliance will be too loose; if they are excessive, it will be too tight. An incorrect fit can determine a potential demineralization of enamel and a bite alteration. Moreover, an incorrect fit can cause a loss of occlusal stability as shown by Freitas et al. [2]. In 2001, Sheridan et al. have demonstrated that the full-time use of retainers for an extensive period will cause significant premature occlusal contacts in the posterior teeth and an anterior open bite [1]. According to the “1-to-3” prosthetic concept, when a full-coverage plastic appliance is seated, the thickness of the appliance between the molars inevitably causes a hinge axis interference and thus an anterior open bite [1, 27]. Butler measured the stability of orthodontic results over the first nine months after the delivery of removable retainers. Sixty percent of the full-time patients lost or broke their retainers, as opposed to 13% of the night-only patients [32]. In a study by Lindauer and Shoff, the thermoplastic retainers were as effective as Hawley retainers in maintaining orthodontic correction, and there was an incidence of an anterior open bite in the Essix group [17, 31, 33].

The thermoformed device is a particular transparent retainer that allows better withstanding wear without causing bite alterations. According to Sheridan et al., when the thermoformed plastic is thin, the premature occlusal contacts can be easily avoided. Also, the Essix retainer is comfortable, fit, and aesthetic, thus guaranteeing the long-term stability of the occlusal alignment [2, 17, 31, 32].

In this article, we want to show how the correct characteristics of the thermoformed appliance can guarantee long-term stability. A clinical case with a 10-year follow-up in which retention was performed with an Essix worn only at night will be shown. Moreover, according to literature thanks to the technical and aesthetic characteristics of Essix, greater long-term stability and patient compliance are enhanced [1, 2, 15, 17, 26–28, 31–37].

## 2. Case Report

**Figure 1:**
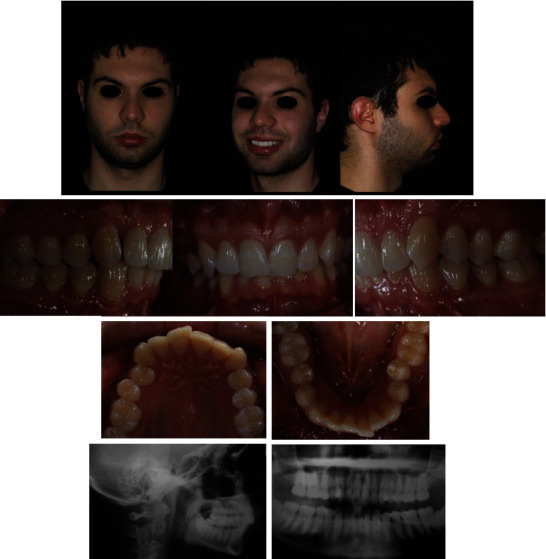
Pretreatment records.

A 33-year-old male patient presented with a class II malocclusion division 2 and normal skeletal pattern. Intraoral examination showed moderate crowding both in the upper arch and in the lower arch. He presented a severe dental and skeletal deep bite (Figure 1). In order to obtain the complete correction of the malocclusion, an extractive treatment plan had been suggested. More precisely, the extraction of the first upper premolars had been proposed. However, the patient refused such therapeutic option by choosing a cosmetic treatment. So, in order to obtain an exclusively aesthetic result, the objectives of the orthodontic treatment were

- to improve the overbite
- to align and level both arches

**Figure 2:**
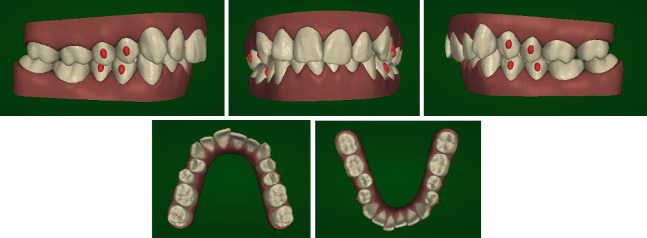
Initial Clincheck.

**Figure 3:**
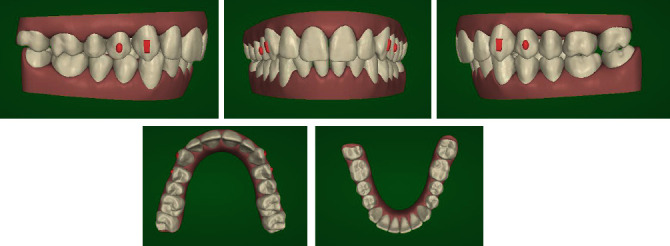
Projection of the treatment outcome.

**Figure 4:**
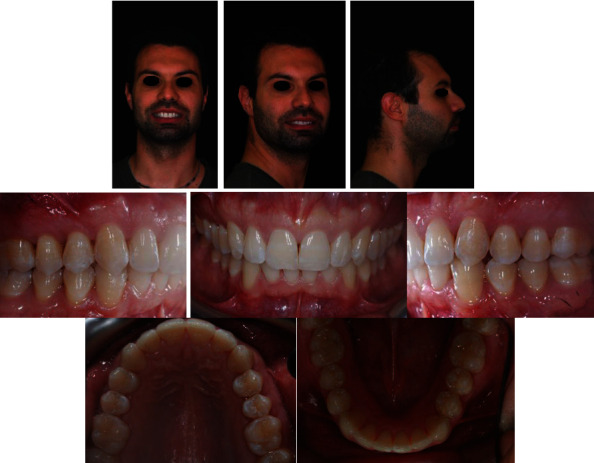
Posttreatment records.

**Figure 5:**
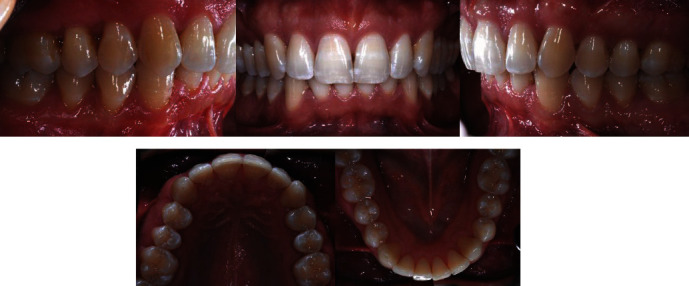
Ten-year follow-up.

According to the patient's desire, a treatment plan was proposed to obtain the aesthetic result of the smile, maintaining the molar and canine class II relationship (Figure 2). The orthodontic therapy was performed using the Clear Aligners System. The Clincheck∗ treatment plan projection anticipated satisfactory resolution of dental anomalies, correction of the overbite, and alignment of the upper and lower anterior teeth (Figure 3). Twenty aligners were planned for the upper arch, and seventeen for the lower arch. The patient was seen every four to six weeks (two to four aligners) to check aligner fit, attachment stability, and cooperation. The initial phase lasted 10 months. The patient required 7 refinement aligners. After 14 months of treatment, the patient was given clear overlay retainers (Figure 4). In this case, it was possible to see a posterior occlusal stability after 10 years. The patient was monitored every year by means of photographic records, showing a correct fit of the thermoformed device without occlusal plane alteration (Figure 5).

## 3. Discussion

Orthodontic treatment has several objectives, and stability of the corrections achieved is one of the most important. There is consensus in the orthodontic literature that some occlusal changes will inevitably occur after treatment. It would be a great benefit to orthodontists to have a detailed prediction of these occlusal changes so that they can be prevented [18, 37, 38]. For this reason, the effects of various diagnostic and treatment factors on occlusal stability in the short and the long term have been extensively investigated [1, 11, 34]. Maintaining the arch form and new position of the teeth after orthodontic treatment is a tedious problem for orthodontists. Both fixed and removable appliances can be used for retention [17, 18, 32, 33, 39].

Essix removable retainers are gaining popularity among orthodontists, thanks to their ability to encapsulate and retain both posterior and anterior teeth. Currently, some clinicians prefer to use the clear removable device called Essix to guarantee the long-term tooth stability after the orthodontic treatment [2, 17, 26, 27, 31, 33]. Because of their minimal thickness and “U”-shaped configuration, transarch stability can sometimes be inadequate.

The removable clear device may raise some problems such as posterior instability and improper long-term fit [17, 26, 28, 33, 35]. However, orthodontists prefer to use the removable retainer because this kind of device has several advantages such as the low cost of fabrication, the minimal bulk and thickness, the durability, and the ease of clearing. [17, 26, 27]

Essix thermoplastic retainers change the rules of permanent retention, because they are thin, but stronger. However, this kind of retainer shows further disadvantages, besides the posterior instability and improper long-term fit: the presence of occlusal contacts and an anterior or lateral open bite [1, 17, 31, 39]. In a study by Littlewood et al., the authors evaluate the long-term stability of 56 patients by randomly assigning Essix and Hawley retainers [16]. The thermoplastic retainers were as effective as the Hawley retainers in maintaining orthodontic correction, but there was an incidence of an anterior open bite in the Essix group. In a prospective RTC, Profitt et al. and Hichens et al. found, after 6 months, that less incisor irregularity occurred in the group of Essix retainers; however, this group had an anterior open bite [35, 36]. In order to reduce the posterior occlusal precontacts, Rinchuse et al. suggest the use of a 3-3 Essix retainer [17]. According to literature, to guarantee long-term stability of occlusal alignment, uttermost cooperation on behalf of the patient is crucial [1, 2, 6, 13–15, 26, 28, 29, 31, 32, 36, 39, 40]. The retainer device must be comfortable, aesthetic, and easy to wear. Only with the above characteristics, full patient compliance is achieved. For these reasons, patients willingly accept to wear a thermoformed appliance for a long time, showing good occlusal stability after 10 years.

## 4. Conclusion

Essix retainers have been proven quite versatile. Their flexibility and positioner effect make them an alternative to spring retainers in correcting minor tooth movements [26, 27].

Orthodontists' concept of retention is moving toward the idea that teeth will move unless retained indefinitely. However, permanent retention implies permanent supervision, and that is where reality clashes with stability [2, 10, 26].

We believe that it is prudent to establish a retention protocol based on the needs and concerns of each individual patient. Therefore, patient cooperation is of the utmost importance [6, 26]. Clinicians prefer to use the clear removable device called Essix to guarantee the long-term tooth stability after the orthodontic treatment [2, 17, 26, 27, 31, 33]. According to literature, in our clinical case, we opted to use a thermoformed appliance with reduced thickness to allow a perfect fit reducing the risk of a posterior open bite [2, 17, 31, 32]. The thermoplastic retainer is comfortable, aesthetic, and easy to wear, and with the above characteristics, full patient compliance can be achieved for a long time. In conclusion, it can be said that thanks to its technical properties, Essix is the effective option to use as a postorthodontic treatment retainer. Its minimum thickness and its ideal fit reduce the risk of a lateral and anterior open bite even after 10 years of follow-up as seen in this case report.

## References

[1] Sheridan J. J., Armbruster P., Moskowitz E., Nguyen P. (2001). Avoiding demineralization and bite alteration from full-coverage plastic appliances. *Journal of Clinical Orthodontics*.

[2] Feritas K. M., Guirro W. J., De Feritas D. S., De Feritas M. R., Janson G. (2017). Relapse of anterior crowding 3 and 33 years postretention. *American Journal of Orthodontics and Dentofacial Orthopedics*.

[3] Yavari J., Shrout M. K., Russell C. M., Hass A. J., Hamilton E. H. (2000). Relapse in Angle class II division 1 malocclusion treated by tandem mechanics without extraction of permanent teeth: a retrospective analysis. *American Journal of Orthodontics*.

[4] Blake M., Bibby K. (1998). Retention and stability: a review of literature. *American Journal of Orthodontics*.

[5] Feritas K. M., Janson G., De Feritas M. R., Pinzan A., Henriques J. F., Pinzan-Vercelino C. R. (2007). Influece of the quality of the finished occlusion on postretention occlusal relapse. *American Journal of Orthodontics and Dentofacial Orthopedics*.

[6] De la Cruz A., Sampson P., Little R. M., Artur J., Shapiro P. A. (1995). Long-term chances in arch form after orthodontic treatment and retention. *American Journal of Orthodontics and Dentofacial Orthopedics*.

[7] Topouzelis N., Diamantidou A. (2003). Orthodontic treatment in patients with reduced periodontium. *Hellenic Orthodontic Review*.

[8] Zachrisson B. U. (1996). Clinical implications of recent orthodontic-periodontic research findings. *Seminars in Orthodontics*.

[9] Case C. S. (1920). Principles of retention in orthodontia. *International Journal of Orthodontia and Oral Surgery (1919)*.

[10] Vanarsdall R. L., White R. P. (1990). Relapse and retention: professional and public attitudes. *American Journal of Orthodontics*.

[11] Sinclair P. M., Little R. M. (1983). Maturation of untreated normal occlusions. *American Journal of Orthodontics*.

[12] Little R. M. (2019). Stability and relapse of dental arch alignment. *British Journal of Orthodontics*.

[13] Little R. M., Riedel R. A., Artur J. (1988). An evaluation of changes in mandibular anterior alignment from 10 to 20 years postretention. *American Journal of Orthodontics and Dentofacial Orthopedics*.

[14] Vaden J. L., Harris E. F., Gardner R. L. (1997). Relapse revisited. *American Journal of Orthodontics and Dentofacial Orthopedics*.

[15] Dyer K. C., Vaden J. L., Harris E. F. (2012). Relapse revisited-again. *American Journal of Orthodontics and Dentofacial Orthopedics*.

[16] Littlewood S. J., Millet D. T., Doubleday B., Bearn D. R., Worthington H. V. (2006). Retention procedures for stabilizing tooth position after treatment with orthodontic braces. *Cochrane Database of Systematic Reviews*.

[17] Rinchuse D. J., Miles P. G., Sheridan J. J. (2007). Orthodontic retention and stability: a clinical perspective. *Journal of Clinical Orthodontics*.

[18] Hawley C. A. (1919). A removable retainer. *Dental Cosmos*.

[19] Gianelly A. A. (2003). Rapid palatal expansion in the absence of cross-bite: added value?. *American Journal of Orthodontics*.

[20] Gianelly A. (2006). Evidence-based therapy: an orthodontic dilemma. *American Journal of Orthodontics*.

[21] Artur J., GArolo J. D., Little R. M. (1996). Long-term stability of mandibular incisors following successful treatment of class II, division, malocclusion. *The Angle Orthodontist*.

[22] Rossouw P. E., Preston C. B., Lombard D. J., Truter J. W. (1993). A longitudinal evaluation of the anterior border of the dentition. *American Journal of Orthodontics*.

[23] Burke S. P., Silvera A. M., Goldsmith L. J., Yancey J. M., Van Stewart A., SCarfe W. C. (1998). A meta-analysis of mandibular intercanine width in treatment and postretention. *The Angle Orthodontist*.

[24] Al Yami E. A., Kuijpers-Jagtman A. M., van’t Hof M. A. (1999). Stability of orthodontic treatment outcome: follow-up until 10 years postretention. *American Journal of Orthodontics and Dentofacial Orthopedics*.

[25] Strang R. H. W. (1949). The fallacy of denture expansion as a treatment procedure. *The Angle Orthodontist*.

[26] Sheridan J. J., Ledoux W., McMinn R. (1993). Essix retainers: fabrication and supervision for permanent retention. *Journal of Clinical Orthodontics*.

[27] Laboda M. (1995). *The effect of Essix appliances on anterior open-bite*.

[28] Hilliard K., Sheridan J. J. (2000). Adjusting Essix appliances at chairside. *Journal of Clinical Orthodontics*.

[29] Sauget E., Covell D. A., Boero R. P., Lieber W. S. (1997). Comparison of occlusal contacts with use of Hawley and clear overlay retainers. *The Angle Orthodontist*.

[30] Knezovic Zlataric D., Celebic A., Valentic-Peruzovic M. (2002). The effect of removable partial dentures on periodontal health of abutment and non-abutment teeth. *Journal of Periodontology*.

[31] Rinchuse D. J., Rinchuse D. J. (1997). Active tooth movement with the Essix appliance. *Journal of Clinical Orthodontics*.

[32] Butler J. (2002). *Assessment of orthodontic stability using an alternative Hawley retainer regimen of “night-time” only wear*.

[33] Lindauer S. J., Shoff R. C. (1998). Comparison of Essix and Hawley retainers. *Journal of Clinical Orthodontics*.

[34] Gunay F., Alpez Oz A. (2018). Clinical effectiveness of 2 orthodontic retainer wires on mandibular arch retention. *American Journal of Orthodontics and Dentofacial Orthopedics*.

[35] Profitt W. R., Fieldes H. W., Sarver D. M. (2014). *Contemporary orthodontics*.

[36] Hichens L. P. Y., Hollinghurst S., Ewings P., Williams A. C. (2006). An RTC comparing the cost-effectiveness between Hawley and vacuum-formed retainers (abstr.). *Journal of Orthodontics*.

[37] Shah A. A. (2003). Postretention changes in mandibular crowding: a review of the literature. *American Journal of Orthodontics and Dentofacial Orthopedics*.

[38] Artur J., Spadafora A. T., Shapiro P. A. (1997). A 3-year follow-up study of various types of orthodontic canine-to-canine retainers. *European Journal of Orthodontics*.

[39] Sheridan J. J., McMinn R., LeDoux W. (1995). Essix thermosealed appliances: various orthodontic uses. *Journal of Clinical Orthodontics*.

[40] Moskowitz E. M., Kaner C. (2004). Predictable retention for the periodontally compromised patient. *Journal of Clinical Orthodontics*.

